# Examining the optimal timing for closed-loop auditory stimulation of slow-wave sleep in young and older adults

**DOI:** 10.1093/sleep/zsz315

**Published:** 2019-12-24

**Authors:** Miguel Navarrete, Jules Schneider, Hong-Viet V Ngo, Mario Valderrama, Alexander J Casson, Penelope A Lewis

**Affiliations:** 1 Cardiff University Brain Research Imaging Centre (CUBRIC), School of Psychology, Cardiff University, Cardiff, UK; 2 School of Biological Sciences, University of Manchester, Manchester, UK; 3 School of Psychology, University of Birmingham, Edgbaston, Birmingham, UK; 4 Department of Biomedical Engineering, University of Los Andes, Bogotá, Colombia; 5 School of Electrical and Electronic Engineering, University of Manchester, Manchester, UK

**Keywords:** closed-loop auditory stimulation, sleep, slow oscillation, age, memory

## Abstract

**Study Objectives:**

Closed-loop auditory stimulation (CLAS) is a method for enhancing slow oscillations (SOs) through the presentation of auditory clicks during sleep. CLAS boosts SOs amplitude and sleep spindle power, but the optimal timing for click delivery remains unclear. Here, we determine the optimal time to present auditory clicks to maximize the enhancement of SO amplitude and spindle likelihood.

**Methods:**

We examined the main factors predicting SO amplitude and sleep spindles in a dataset of 21 young and 17 older subjects. The participants received CLAS during slow-wave-sleep in two experimental conditions: sham and auditory stimulation. Post-stimulus SOs and spindles were evaluated according to the click phase on the SOs and compared between and within conditions.

**Results:**

We revealed that auditory clicks applied anywhere on the positive portion of the SO increased SO amplitudes and spindle likelihood, although the interval of opportunity was shorter in the older group. For both groups, analyses showed that the optimal timing for click delivery is close to the SO peak phase. Click phase on the SO wave was the main factor determining the impact of auditory stimulation on spindle likelihood for young subjects, whereas for older participants, the temporal lag since the last spindle was a better predictor of spindle likelihood.

**Conclusions:**

Our data suggest that CLAS can more effectively boost SOs during specific phase windows, and these differ between young and older participants. It is possible that this is due to the fluctuation of sensory inputs modulated by the thalamocortical networks during the SO.

**Statement of significance**

Sleep slow oscillations (SOs) and spindles are critical for memory and restorative functions. Concurrent findings have demonstrated that phase-locked auditory stimulation boosts SO waves and spindles while enhancing associated cognitive and physiological processes. Nevertheless, it is not clear how precisely the stimulus should be applied to maximize the response of these sleep patterns. Here we determine the SO phase intervals where auditory clicks produce larger SO amplitudes and increased spindle likelihood in young and older adults. These results suggest distinctive processes for SO and spindle enhancement, stressing the effects of aging in response to the stimulation. These findings also provide further grounds for developing and improving stimulation techniques to modulate these sleep patterns in both clinical and research applications.

## Introduction

The use of non-pharmacological and non-invasive techniques to enhance slow-wave sleep (SWS) is a rapidly growing research area. SWS is characterized by slow-wave activity (SWA) which are bursts of high amplitude slow oscillations (SOs) at 0.5 to 2 Hz [1]. SWA predicts overnight memory consolidation [2], it is tightly linked to immune function [3] and to the neurotoxic waste clearance such as β-amyloids [4]. However, these oscillations decrease across the lifespan [5, 6], and this gradual loss of slow waves is characterized by increased fragmentation of sleep and decreased cognitive function [7]. Therefore, several authors suggest that this reduction in SWA leaves the older adult at increasing risk of physiological decline [8, 9].

Transcranial magnetic [10] and electric [11, 12] stimulation have demonstrated to enhance SWA, but closed-loop auditory stimulation (CLAS) has been confirmed as a promising technique since this is non-invasive, inexpensive, easy to apply and effective [13]. In CLAS, an acoustic click is applied during the peaks of SOs, producing an increase in the amplitude of the ongoing wave and in fast spindle activity during the period of stimulation [14]. This technique can increase overnight memory retention for a word-pair matching task in young and older adults [14, 15], it has demonstrated to boost hippocampal activity [16], impact on immune processing [3] and have a positive effect on the autonomic function in sleep [17]. However, this method fails to increase the time spent in SWS or the number of detectable SOs across the total night [13, 14]. Also, CLAS appears not to affect the consolidation of other memory tests such as finger tapping, picture memory or memory of names and faces [18].

Importantly, CLAS is still relatively new and has yet to be optimized. For instance, it has been suggested that stimulation should be applied during particular timings of the slow wave to obtain an increased and favorable cortical response [19–21], but there is no validation on which timing is the best. Similarly, although one study did show that CLAS can successfully enhance both SWA and memory consolidation in older adults [15], older adults show shallower SOs that may require different optimization parameters for stimulation than younger adults. Due to the intrinsic differences between the SWS in these two groups, and the medical relevance of improving sleep in healthy older adults, we sought to characterize optimal stimulation windows for both groups. Specifically, in this study, we aimed to identify the SO phase at which a click stimulus maximally enhances the slow-wave trough amplitude and the SO phase at which clicks maximally increase spindle likelihood, amplitude and duration.

## Materials and Methods

### Datasets and experimental procedures

Polysomnographic data, including EEG and hypnogram of 38 individuals, were obtained from three independent datasets. All protocols were approved by the appropriate ethic committees of local institutions (University of Lübeck, University of Tübingen, and University of Los Andes) and written consents were obtained for each participant. Participants in all datasets were healthy non-smokers with good hearing, regular sleep/wake patterns and no medications known to affect sleep. Likewise, subjects were asked to avoid taking naps and to consume alcohol and caffeine drinks in the day before the experiments. Experimental procedures were carried out in University of Lübeck in Germany (uLub, *N* = 11, 8 females, and mean ± SD age = 24.2 ± 3.0 years; data previously published [13]), University of Los Andes in Colombia (uAnd, *N* = 10, 6 females, and mean ± SD age = 27 ± 5.5 years) and University of Tübingen in Germany (uTub, *N* = 17, 9 females, and mean ± SD age = 55 ± 5.0 years). In the three research studies, the participants spent two experimental nights in the laboratory undergoing one experimental stimulation (STIM) and one no-stimulation condition (SHAM). The order of experimental conditions was balanced across subjects and separated by at least one week. The data was divided in young adults (ages < 40 years in uLub and uAnd datasets) and older middle-aged subjects (ages > 50 years; uTub dataset) and analyzed accordingly (Table 1).

**Table 1.** Demographic and sleep summaries of each dataset

	uLub (young)			uAnd (young)			Young (uLub + uAnd)			Older (uTub)		
Subjects	10			11			21			17		
Sex												
Female	72.7%			60.0%			66.7%			52.9%		
Age (years)												
Mean	24.2			27.0			25.7			55.0		
SD	3.0			5.5			4.7			5.0		
	STIM	SHAM	*p*	STIM	SHAM	*p*	STIM	SHAM	*p*	STIM	SHAM	*p*
*Sleep architecture*												
Total sleep time (h)												
Mean	7.0	7.0	.84	6.4	6.5	.71	6.7	6.8	.70	7.7	7.6	.95
SD	0.4	0.2		0.8	0.6		0.7	0.5		0.6	0.7	
N1 sleep (%)												
Mean	7.6	5.5	.09	3.9	4.0	.96	5.8	4.8	.40	4.5	4.9	.54
SD	2.9	2.4		5.1	3.9		4.4	3.2		1.7	2.0	
N2 sleep (%)												
Mean	45.0	46.7	.56	37.5	36.5	.72	41.4	41.8	.87	50.3	51.5	.75
SD	7.9	5.4		6.6	5.9		8.1	7.6		10.6	9.5	
N3 sleep (%)												
Mean	19.1	18.0	.70	33.1	30.5	.53	25.8	23.9	.56	8.4	6.9	.43
SD	7.0	6.2		9.4	9.1		10.8	9.9		6.4	4.9	
REM sleep (%)												
Mean	14.0	16.9	.17	20.9	22.7	.53	17.2	19.7	.23	15.8	16.1	.91
SD	4.2	5.4		7.1	6.1		6.6	6.4		6.5	7.7	
Arousals (N)												
Mean	65.1	55.9	.11	28.5	24.3	.57	47.7	53.8	.33	49.2	53.8	.59
SD	16.1	7.9		20.9	8.6		22.3	26.1		22.3	26.1	
^†^SO (µV) in D.P												
Mean	−103.8	−98.5	.28	−92.4	−87.9	.31	−98.4	−93.4	.18	−79.8	−79.1	.83
SD	12.1	10.4		10.2	9.2		12.4	11.0		9.2	8.7	
Total trials												
Mean	324.2	736.6	**<.001**	354.9	423.7	.57	338.8	587.6	**<.01**	237.2	605.3	**<.001**
SD	170.8	275.0		238.6	291.7		201.1	319.0		177.1	292.6	

Macrostructure of sleep did not show significant differences between stimulation conditions. The uLub and uAnd datasets were considered as the Young cohort group, whereas the older cohort is composed by the uTub dataset. *SD*: Standard deviation; *uLub:* University of Lübeck dataset; *uAnd:* University of Los Andes dataset; *uTub*: University of Tübingen dataset. (†) SO trough amplitude. (D.P) Detection period. Values in bold are statistically significance at alpha = 0.05.

### Sleep monitoring

Standard polysomnography consisting of EEG, chin EMG and EOG were continuously recorded with either a BrainAmp DC amplifier (Brain Products), or with a LTM64 amplifier (Micromed). Electrodes were positioned according to the international 10–20 system and referenced to an average of the two mastoids (M1, M2). Common channels across the three datasets comprised the following scalp electrodes: F3, Fz, F4, C3, Cz, C4, P3, Pz, and P4. Ag/AgCl electrodes were used, and impedances were always kept below 5 kΩ. The data were sampled at 500 Hz for uLub and uTub datasets; and at 256 Hz in the uAnd dataset and saved without further filtering in uAnd and uTub datasets, whereas uLub signals were saved and filtered between 0.03 and 150 Hz.

### Auditory closed-loop stimulation

The three datasets applied a similar detection and stimulation algorithm, as described in the original study by Ngo et al. [13]. Custom-made real-time automatic detection algorithms were applied to an acquired signal from a frontal channel (Fz in uLub, F3 in uAnd, and AFz in uTub) throughout a close-loop stimulation interface independent of the recording system (“Power1401 mk 2” data acquisition interface for uLub and uTub whereas this was developed in MATLAB for uAnd).

**Figure 1.**
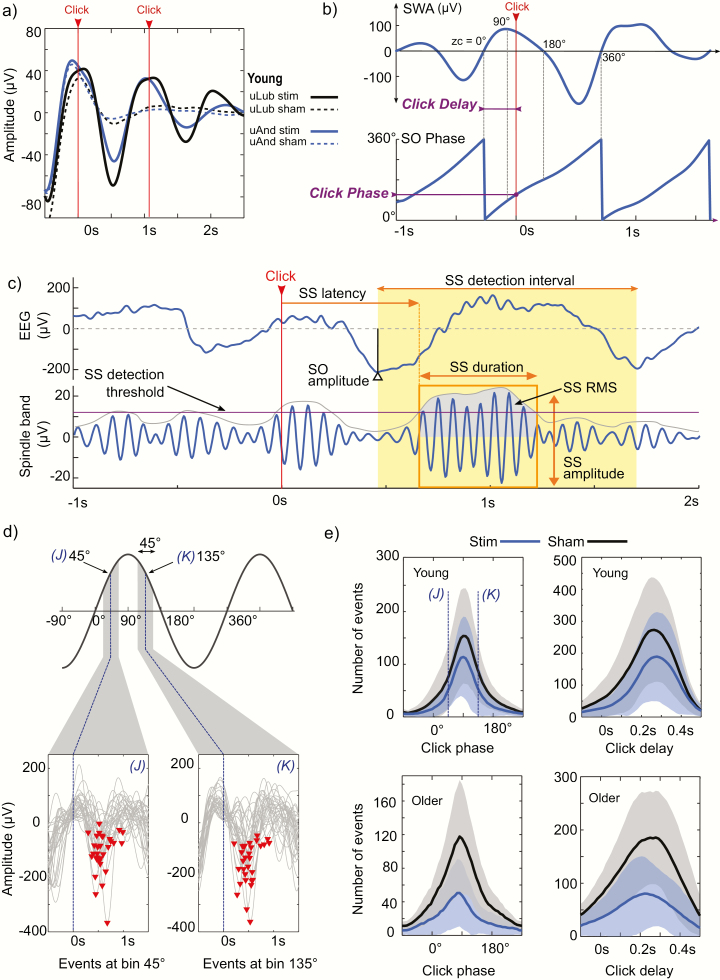
Description of datasets and analysis methods. (a) Stimulation protocols were similar in all datasets. Two clicks marked by the vertical arrow lines were presented in the predicted up-state. All datasets presented similar ERPs with increased SOs amplitude for the STIM condition. (b) The delay from the zero-crossing to the click time (vertical arrow line) and the corresponding click phase were obtained and used as reference points for further analysis. For SO wave phase, 0° states the negative to positive zero-crossing (ZC), −90° and 270° represent the SO troughs and 90° the SO peak of the trace. (c) Detection of sleep spindles (SS) as well as SO and SS measurements used in the analysis. Only SS that start in the detection interval were analyzed (yellow shadow). (d) For the detected and stimulated SOs, the trough-to-trough interval in both phase and time was divided into 50 bins. For instance, in the phase analysis, trials in which the stimulation was applied 45° around the bin centre were selected and comprise the events of each bin. Here we show 30 events of one young subject around the 45° bin *(J)* and 135° bin *(K)*. (e) Histograms of detected events for all datasets. As CLAS targets SO peaks, the distribution of events is not even across all bins, 45° bin *(J)* and 135° bin *(K)* are also depicted for reference. Shaded areas represent subject mean ± SD.

The same protocol of stimulation was applied in all datasets. Acoustic stimuli for the STIM condition consisted of stereophonic clicks of pink noise (50 ms duration) with rising and falling slopes (5 ms duration), and stimulation timestamps were recorded online when clicks were applied. For the SHAM condition, the detection protocol was identical to STIM, but the sound was muted. The streamed signal was filtered in the SO frequency band and negative EEG deflections that surpassed an adaptative threshold were identified indicating a SO down-state during SWS. Briefly, for the uLub dataset, the adaptive default threshold was set to −80 µV and was then updated each 0.5 second to the largest negative value from the preceding 5-second interval if that value was lower than the default. A similar method was used in uTub dataset, but the default threshold was set to −80 µV for most subjects and adjusted to −60 µV and −50 µV for two subjects. For the uAnd dataset, the adaptive default threshold was set to −60 µV and was then updated to half the amplitude of the detected trough if that value was more negative than the default. After each trough detection, two consecutive auditory clicks were applied on subsequent SOs at times defined individually by each algorithm and specifying a stimulation trial (the trial inter-stimulus interval for consecutive clicks was 1.076 seconds for uLub, 1.053 ± 0.06 seconds for uAnd, and 1.096 ± 0.09 seconds for uTub). After each trial, there was a pause for 2.5 seconds before trough detection was resumed. Stimulation was applied during sustained non-rapid eye movement sleep (NREM), including N2 and N3 stages, and this was manually halted if there was evidence of an arousal or REM. Specifically, detection of SOs for stimulation was started manually once participants had spent at least 5 contiguous minutes in NREM (N2 or N3), and then continued for 210 minutes in uLub and uTub datasets and for 250 minutes in uAnd dataset. Stimulation was re-started when N2 or N3 resumed. Periods without stimulation did not count towards the time of the stimulation period. The datasets, collected in different institutions, produced similar event-related potentials (ERP) responses and the same STIM vs. SHAM responses, as seen in Figure 1a.

### Sleep and EEG analysis

Sleep scoring was performed according to ASSM scoring criteria [1] by two trained experimenters blinded to stimulation conditions. All artifacts and arousals were marked in the hypnogram. The total time spent in sleep was computed from the first transition to any sleep stage from wake until the last transition from any sleep stage to wake (Table 1). We focused on events detected in Fz for SOs and Cz for sleep spindles (SS) because these are the locations where those events are more pronounced [1]. The raw data were resampled at 200 Hz with linear interpolation after applying an antialiasing low-pass FIR filter.

For the primary analyses, only marked stimulations during N3 sleep stage were retained. Because CLAS is a self-limiting process to repetitive stimulation [14], and to avoid confusion caused by responses to the second click, we chose our analysis window to include only responses to the first click of each trial. Click stimuli that overlapped with an arousal or artifact were removed. SWA was obtained from the EEG signals filtered between 0.5–2 Hz using a zero-phase windowed equiripple FIR filter (3 dB at 0.25 and 3.08 Hz; >37 dB at *f* < 0.01 Hz and *f* > 4 Hz). Only waves were considered as SOs when their negative deflection had consecutive zero crossings between 0.25 to 1.0 seconds, regardless of the wave amplitude [22]. The response of the SWA to the auditory click was evaluated as the absolute amplitude of the subsequent trough following stimulation; therefore, all trials where the second stimulation was placed before this minimum were excluded from the analysis.

Spindle activity was determined by applying a zero-phase bandpass FIR filter between 11 and 16 Hz (3 dB at 10.62 and 17.38 Hz; >40 dB at *f* < 10.01 Hz and *f* > 18 Hz). Then, the root mean squared (RMS) was computed by using a time window of 0.2 second [23]. Candidate SS events were first detected as those discrete events where RMS signal surpassed a threshold established as the 86.64 percentile (equivalent to 1.5 SD over the mean for a Gaussian distribution) of the spindle activity during N3. Then, SS were identified as events with duration between 0.3 and 3 seconds [24], with at least five oscillations, a unimodal peak in the spindle frequency band (11–16 Hz) and decreasing power for higher frequencies computed by the Morlet wavelet [25]. Furthermore, as previous studies have shown that spindle activity is locked to the SO following the stimulus [13, 14, 26], we defined stimuli-dependent SS as those events that began in the trough-to-trough interval after the marked click. Finally, targeted measures used for further analysis were obtained as indicated in Figure 1c as: the absolute value of SO trough amplitude (SO amplitude), the SS likelihood (from stimuli-dependent SS), the SS latency (time from click to the beginning of SS event), SS duration (time of SS above the detection threshold), SS RMS (root mean squared of SS) and SS amplitude (largest peak-to-peak amplitude of the filtered signal).

### Time- and phase-locking analysis

Time-locked analysis was defined for the delay between the time of the acoustic click and the negative-to-positive zero-crossing (ZC) of the stimulated SO (click delay) (Figure 1b). This time was chosen as the reference point because it is independent of the time between the trough detection and the time of stimulus. In each of the recordings, a trace of the time-dependent response was obtained by pooling the discrete values of the targeted measures into windows of 200 ms in 50 bins ranging from −100 to 600 ms, regardless of the temporal location of a given trial in the overall recording. This created a series of bins with overlapping information containing the ensemble of targeted measure values for the independent trials around the bin center. Likewise, this subdivision is made under the assumption that cortical response does not change drastically for small time or phase differences.

Phase-locked analysis was based on the SO phase, where the stimulus was applied (Figure 1b). Slow-wave phase was obtained across the night from the analytic signal composed by the real signal in the SWA band and its imaginary component, computed as the representation of the real signal shifted by a quarter of cycle by a Hilbert transformer filter [27]. The Hilbert filter was designed as a least-squares linear-phase FIR filter, with unitary gain through the entire SWA band and 250 ms delay. This procedure allowed us to obtain the phase of slow waves based on the past conditions of the signal and minimizing the effect of the stimulus in the phase calculation. For each slow wave selected as response to the stimulation, an additional individual analysis was performed to determine whether the phase cycle of the analytic signal corresponded to a trough-to-trough cycle in the SWA filtered trace. Because of the broad band of the SWA, the signal may be composed by multicomponent frequencies in some individual SOs (i.e. multiple peaks or troughs in half SO cycle) [27, 28]. Nevertheless, no further filtering was applied as we were interested in keeping the temporal features of the slow wave. Hence, for those slow waves where the signal does not show the narrow band behavior due to the presence of multicomponent frequencies (multiple frequencies at the same timestamp), the phase trace was fitted to the polynomial (<5th order) which minimizes the squared error with non-decreasing slope in the trough-to-trough cycle. As for the time-locked case, a trace of the phase-dependent response was obtained for each recording by pooling the discrete values of the targeted measures of all trials into windows of 45° for all the circular range in 50 bins, where –90° and 270° correspond to the slow-wave troughs, 90° to the extreme positive phase and 0° to the negative to positive zero crossing (Figure 1, b and c). For the phase response, this procedure created a series of bins of angular data with overlapping information containing the response of grouped trials to the phase of auditory stimulation (Figure 1c). Similar results were found by dividing the wave range in different bin quantity for both time-locked and phase-locked analysis.

To further quantify phase-dependency of the after-stimulus response, a pairwise response index (PRI) was computed for each subject. This was computed as the proportion of pairwise bin comparisons in which a determined delay/phase bin has a higher statistic compared to the others, as described by

$$\begin{array}{l} PRI_{\left(n\right)}=\;\;\;\frac{1}{N}\underset{j=1}{\overset{N}{\sum }}\;sign\left(t_{n,j}\right) \end{array}$$

where ***n*** is the delay/phase bin of interest, ***N*** is the number of analyzed pairwise comparisons for this bin and ***t***_***n,j***_ corresponds to computed Welch *t-*values for the ***j*** pairwise comparison of bin ***n***. In this way, the pairwise response index will have a value of 1 if the effect of the stimulus for a delay/phase-bin is consistently larger for all comparisons, whereas a value of −1 indicates a delay/phase-bin with steady lower effect after stimulus.

### Statistics

Significant differences between STIM vs. SHAM conditions for pairwise comparisons across subjects were obtained using the Welch unequal variance *t*-test with the Moser–Stevens correction for degrees of freedom. Pairwise comparisons of angular data were calculated using a circular one-way ANOVA for circular data with the Watson–Williams Test [29]. A Benjamini-Yekutieli procedure for controlling the false discovery rate (FDR) was applied to correct for multiple comparisons [30]. Unless otherwise noted, statistics were computed in MATLAB.

The statistical evaluation of time-locked and phase-locked analyses examine the incidence of acoustic stimuli on two aspects: (1) associated delay/phase-dependent differences between experimental conditions, and (2) delay/phase-dependency of the preeminent response to auditory stimuli. Nevertheless, as the direct comparison of measures of interest may lack statistical power in some bins with only a small number of events, we then introduced a Monte Carlo simulation to compare the values of each bin, increasing statistical power and balancing the effect size across bins. The process of this analysis is explained below.

Firstly, to evaluate differences between conditions of targeted measures in similar delay/phase bin, the descriptive statistics of the set of values of each bin of the SHAM condition were extracted and fitted to a Gaussian model, which defines the spontaneous dynamics of the targeted measure. Then, a simulated distribution of the fitted model was generated using 200 realizations and established as the Monte Carlo set for the corresponding bin (MC). Next, all pairwise distances computed as the subtraction of values were obtained for SHAM vs. Monte Carlo (SHAM—MC) and STIM vs. Monte Carlo (STIM—MC), thereby obtaining a distribution of distances characteristic of each bin for both cases. Subsequently, the average distance for each delay/phase-dependent bin is computed for each subject and used as the representative statistic for SHAM vs. STIM comparisons. Similarly, for the evaluation of binomial responses in between condition analysis, the event likelihood for each delay/phase bin was assessed by computing the geometric mean of the event ratio from 200 realizations using randomization of two-thirds of the events of each bin.

Secondly, to study the main response to the acoustic stimulus, pairwise comparisons were computed, and *t*-values obtained within all the delay/phase bins. For this, a distribution was obtained for each delay/phase bin by computing the mean of 200 realizations using a randomization of two-thirds of the events of each bin. Then, bin differences from the mean distributions were assessed using the Welch unequal variance *t*-test. For all the methods described above, the representative bin statistics are equivalent within all pairwise comparisons while maintaining equivalent average of original bin distributions and increasing the power of paired comparisons without further assumptions.

Finally, we implemented a binary logistic regression analysis to evaluate the SS likelihood from SS lag and stimulation phase. This model describes a generalized linear model with a logit link function using explanatory variables that have been previously reported to affect the SS outcome after stimulation. These variables are the SS lag (*Lag*) [31] and the SO phase when the auditory click was applied [13]. Additionally, as the information of stimulus phases is a circular predictor, we included the sine and cosine of stimulation phase as predictor variables instead of the phase value itself to accommodate the circular covariate [32, 33]. Then, as a circular-linear regression model, the regression coefficients of the circular covariates allow us to determine the acrophase angle which indicates the angle where the log odd reaches its highest value [34, 35]. These binary logistic regression analyses were computed using SPSS.

## Results

### Overview of the datasets

No differences were found in the sleep architecture between experimental conditions within datasets. Twenty-one subjects from the combined uLub and uAnd datasets comprise the young group, whereas 17 subjects from the uTub dataset include the older group for analysis. There was no significant difference in the sleep architecture for the STIM vs. SHAM conditions in all datasets (all *p* > .05). The same result was found for datasets in the young group, where no differences in sleep architecture were found when they were evaluated either individually or altogether. For the total number of analyzed events, the quantity of SHAM trials is higher than STIM trials in both young and older datasets (Table 1). Likewise, the mean number of events in SHAM is consistently higher than STIM across all delay/phase bins as seen in Figure 1e. These differences in number of trials between SHAM and STIM conditions may be due to refractory periods caused by continuous auditory stimulation [14], affecting the number of online detected SO.

Together the adaptive threshold for SO trough detection and the period of stimulation might influence subsequent enhancement. Therefore, we compared the pre-stimulus SO down-states amplitudes between the two stimulation conditions (SHAM vs. STIM) to check that the criteria for SO detections were similar in both cases. Also, we divided each subject’s detection period in three equally spaced blocks according the time of stimulation (early, middle, and late) and compared in the SHAM vs. STIM conditions in each block. We ran a Two-way repeated measures ANOVA to examine the effects of stimulation condition (SHAM vs. STIM) and time period (early, middle, and late) on the amplitude of detected SO down-states. The statistical analysis revealed no differences in detection of SOs for any dataset across stimulation condition (uLub: *F*_1,10_ =3.32, *p* = .098, ηp^2^ = .25; uAnd: *F*_1,9_ =3.27, *p* = .581, ηp^2^ = .03; uTub: *F*_1,16_ =2.49, *p* = .134, ηp^2^ = .13), no differences regarding the stimulation period (uLub: *F*_1,20_ =1.56, *p* = .242, ηp^2^ = .13; uAnd: *F*_1,18_ = 2.36, *p* = .125, ηp^2^ = .21; uTub: *F*_1,32_ = 0.70, *p* = .505, ηp^2^ = .04), and no interaction between stimulation condition and the detection period (uLub: *F*_2,20_ =1.57, *p* = .826, ηp^2^ = .01; uAnd: *F*_2,18_ = 3.89, *p* = .648, ηp^2^ = .04; *F*_2,32_ = 1.68, *p* = .202, ηp^2^ = .09). Furthermore, while the thresholds were lower in the older group in absolute terms of microvolts, examining these values relative to the mean amplitude of all troughs during the detection period shows that thresholds were actually 2.54% more demanding for the older than for the younger cohort (µ ± SD, Old = 95.4% ± 18.8%, Young = 92.8% ± 3.8%; *t*(60.4) = 3.82, *p* < .001; 95% CI = 1.21% to 9.87%). Thus, in both protocols, the detected troughs were greater than 90% of the amplitudes within the same period of stimulation. This indicates that in both cohorts, the algorithm of detection was consistently selecting the largest waves for stimulation. Therefore, we can infer that detected SO waves are on average comparable during the time of detection, and there was no bias by the adaptive SO detection thresholding.

### SOs respond to auditory stimulation throughout the upstate in young adults but during a shorter interval in older adults

**Figure 2.**
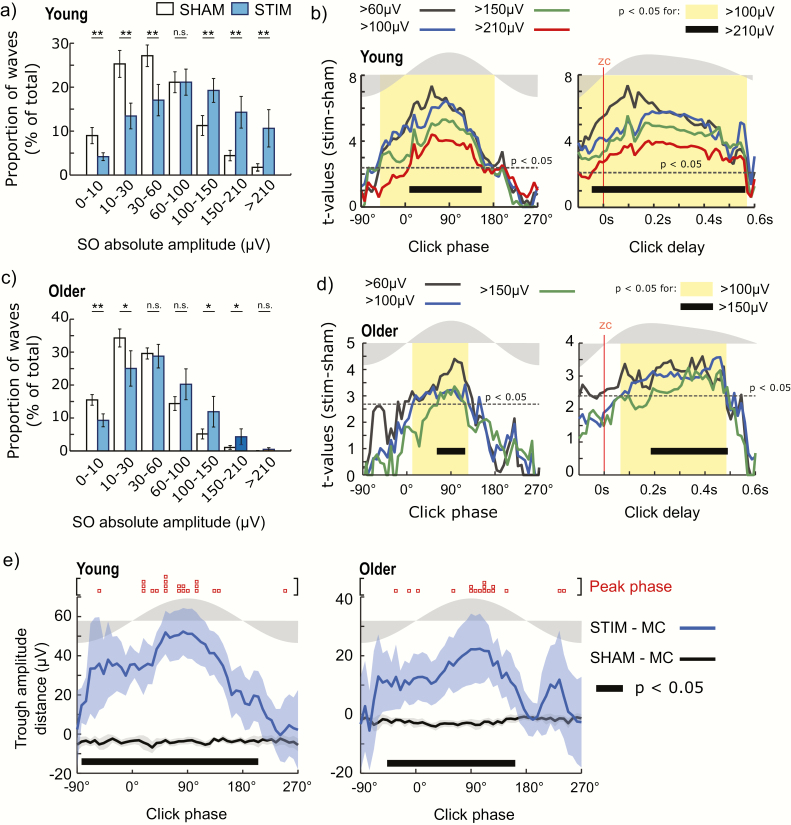
Slow-wave amplitudes after CLAS in young and old subjects. (a) Distribution of the post-stimulus absolute trough amplitude of SOs during SHAM (white bars) and STIM (blue bars) in young subjects. (b) Size of the difference between STIM and SHAM for post-stimulus SO amplitudes for clicks applied at different phase/delay-bins for young people. Dotted line represents the significance threshold for *p* < .05 after FDR correction. Yellow shadow indicates intervals for which a significant increasing of >100 µV amplitudes was found. Click delay was evaluated from the negative to positive zero-crossing (zc). For reference, the gray shading on top of the axes displays the morphology of the average SO wave. (c) Distribution of the post-stimulus trough amplitude of slow waves during SHAM and STIM conditions for older subjects. (d) Size of the difference between STIM and SHAM for post-stimulus trough amplitudes for clicks applied at different phase/delay-bins for older people. (e) Absolute distance (mean ± 95% CI) of post-stimulus trough amplitudes to spontaneous activity over different phase bins for SHAM vs. Monte Carlo (SHAM—MC) and STIM vs. Monte Carlo (STIM—MC). Thick black bars show phase intervals in which significant differences were observed at *p* < .05 after FDR correction. Red squares remark maximal significant distance for each subject after corrected *p* < .05. (*) *p* < .05 and (**) *p* < .01 after FDR correction; (n.s.) for nonsignificant.

To assess the general slow-wave response to CLAS, we first evaluated the absolute amplitude value of the post-stimulus SO trough for both stimulation and sham conditions collapsed across all stimuli timing ranges. As expected, the proportion of lower amplitude events (<60 μV) was shifted towards larger amplitudes (>100 μV) for young adults (Figure 2a, *p* < .05 after FDR correction) in the stimulation condition compared to sham. To examine the relevant timing of stimulation, we performed a between condition analysis for all stimulated phases. Figure 2b indicates that stimulation throughout both the late negative deflection and the entire positive wave results in increased slow-wave amplitude in young subjects (>100 μV in the range −53.2° to 177.9°). Nevertheless, the proportion of higher amplitude post-stimulus events increases when clicks are delivered on the positive slow-wave phase. This is shown in Figure 2b for post-stimulus troughs with absolute amplitudes higher than 210 μV (in the phase range 6.9° to 156.9°). As seen in Figure 2e, post-stimulus slow waves were on average 39.6 μV (95% CI = 30.5 μV to 48.7 μV) bigger than spontaneous slow waves when a click was applied within the most effective interval (e.g. the interval where stimulation made a significant difference) (*p* < .05 after FDR correction for the phase interval −82.8° to 205.2°). Post-stimulus events had higher amplitudes when the stimulus was applied before the SO peak; hence, the phase which represents the maximal distance for trough amplitude in the STIM vs. SHAM comparison across young subjects was 66.4° (95% CI = 42.1° to 90.7°) (Figure 2e left, peak phase).

Interestingly, the effect of auditory stimulation on the event-related trough amplitude is notably reduced in older adults. For the older population, the proportion the post-stimulus high amplitude events slightly increases for post-stimulus trough events in the 100–210 μV interval reinforced by the reduction of lower amplitude events (<30 μV) although the outcome of stimulation was not as strong as in younger subjects (Figure 2c, >100 μV in the range 9.3° to 129.6°, *p* < .05 after FDR correction). Additionally, in older subjects, the post-stimulus troughs averaged just 18.3 μV (95% CI = 11.1 to 25.6 μV) more than for clicks applied during the optimal stimulation interval (e.g. the interval of significant difference between STIM and SHAM) (*p* < .05 after FDR correction for the phase interval −39.6° to 162°). This indicates that older subjects have a much lower effect of stimulus than young subjects (two-tailed Welch *t*-test, *t*(35.6) = 3.6, *p* < .001). Furthermore, unlike the younger group, SO troughs in older subjects had higher amplitudes when the stimulus was applied later at the phase 83.6° (95% CI = 45.2° to 122.1°; Figure 2e, right, peak phase). However, at the group level, there was no individual phase that was significantly different between young and older subjects (Watson–Williams Test, *F*_1,36_ = 2.91; *p* = .097).

### Click stimulation near the peak elicits a larger SO response

To ensure that the significant findings in our above analyses (STIM vs. SHAM) were not confounded by a basic difference in excitability (i.e. responses are always greater in STIM than SHAM), we performed a control analysis comparing responses within-subject. Observation of similar results in both this and the STIM vs. SHAM comparison (shown above) will strengthen confidence in the overall findings.

**Figure 3.**
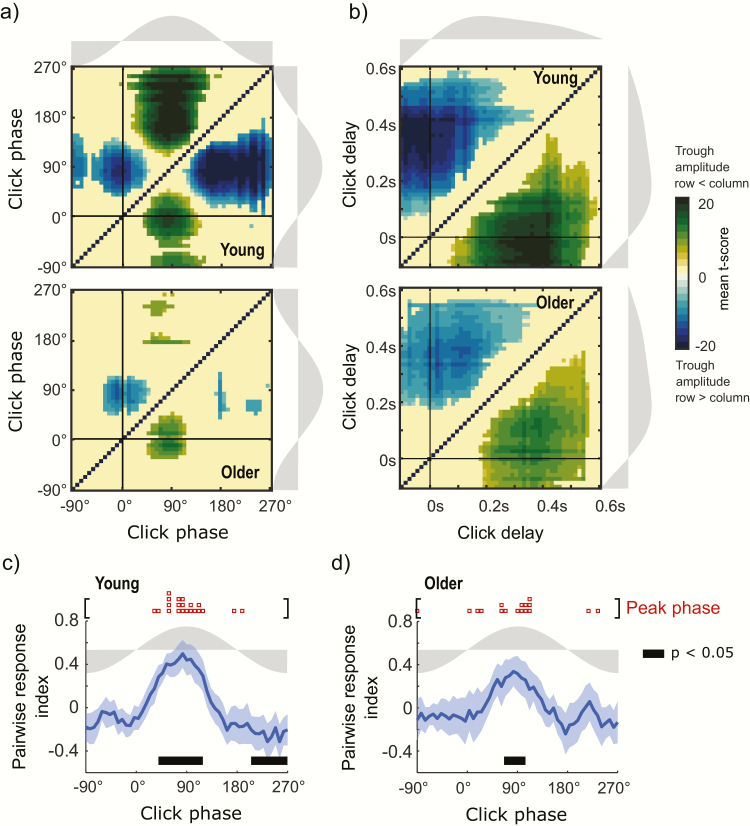
Pairwise comparison for SO trough amplitude in STIM condition. (a, b) Pairwise comparison matrix for post-stimulus trough amplitude events evaluated in click phase and click delay bins for young (a) and older subjects (b). X and Y axis represent the same bins comparing each point to every other point. Matrix values correspond to the mean size of the difference (t-values) of bin pairwise comparisons significant after FDR correction. (c, d) Pairwise response index (mean ± 95% CI) for click phases for young (c) and older subjects (d). Thick black bars show phase intervals in which significant differences were observed at *p* < .05 after FDR correction.

Figure 3a shows the result of the within condition analysis for STIM for young and older subjects. Specifically, this figure shows the average group-level *t*-values where comparisons are significant after FDR correction. Color coding shows which phase-bins differ significantly in the post-stimulus trough amplitude. Thus, for young subjects, stimulations applied between 40° and 140° generate larger amplitude SO responses than stimuli applied during other phases (<39.6° and > 140°). Likewise, for older subjects, stimulation near the peak, e.g. between 46.8° and 118.8°, showed stronger responses than stimulation on the rising slope of the positive wave, e.g. between −46.8° and 39.6°. Figure 3b also shows equivalent pairwise comparisons for the delay analysis. This demonstrates that responses were maximal when stimulation occurred between 100 ms and 550 ms after the zero-crossing (young: 82 to 560 ms; older: 194 to 558 ms), whereas lower responses were shown for clicks applied on the first section of the rising slope (young: <54 ms; older: <166 ms after the negative to positive zero crossing).

To further specify the wave phase where stimulation has a more pronounced effect, we computed the pairwise response index (PRI) for each subject and computed differences at the group level. The PRI is a measure of what proportion of comparisons were positive or negative (see Methods). For younger subjects, the PRI is maximal for clicks applied in phases between 39.6° and 118.8° (mean phase = 87.2°, 95% CI = 69.3° to 105.1°) and minimal for clicks > 205.2° (*p* < .05 after FDR correction, Figure 3c). For older subjects, the maximal effect was found between 68.4° and 104.4° (*p* < .05 after FDR correction; mean phase = 83.6°, 95% CI = 45.2° to 122.1°, Figure 3d). Despite this apparent age-related difference, and as seen in the between condition analysis reported in the section above, the circular one-way ANOVA showed no difference among groups for the PRI optimal click phase (Watson–Williams Test, *F*_1,36_ = 0.04; *p* = .841).

In summary, both between condition and within condition analyses show that the absolute post-stimulus amplitude is modulated by the SO phase where a click is applied. Although reduced for the older group, there is a wide interval of excitability where the amplitude of post-stimulus troughs is increased by the click in both young and older subjects. This interval is contained mostly in the rising slope of the SO and its positive half wave. Nevertheless, maximal stimulation effects were found for clicks applied around the peak wave. Importantly, results from both the within and the between condition analyses were highly compatible since the phases of maximal response were very similar in both cases (Figure 2). Auditory stimulation primarily impacted high amplitude trough events in both young and old participants (>210 μV for young and >100 μV for older subjects in Figure 2, b and d) within this maximally responsive window.

### Spindle activity is strongly influenced by stimuli applied to the positive slope of slow waves

**Figure 4.**
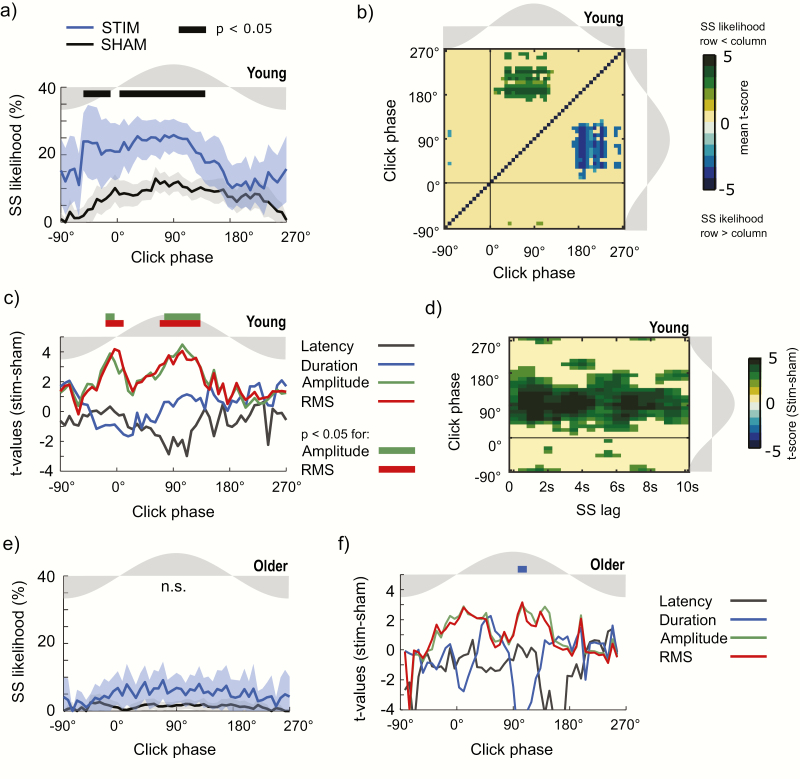
Sleep spindle response to CLAS. (a) sleep spindles (SS) likelihood (mean ± 95% CI) modulated for the click phase for STIM and SHAM conditions for young subjects. (b) Pairwise comparison matrix for post-stimulus SS likelihood for young subjects. Matrix values correspond to the mean size of the difference (*t*-values) of bin pairwise comparisons significant after FDR correction. (c) Modulation of SS features depending on the click phase for STIM and SHAM conditions for young subjects. (d) Size of the difference between STIM and SHAM conditions for significant *t*-values after FDR correction indicating SS likelihood as a joint function of the click phase and the SS lag for young subjects. (e) SS likelihood (mean ± 95% CI) modulated for the click phase for STIM and SHAM conditions for older subjects. (f) Modulation of SS features depending on the click phase for STIM and SHAM conditions for older subjects. Thick horizontal lines on the top display phase intervals in which conditions differed at *p* < .05 after FDR correction.

Prior work [13, 15] has shown that CLAS can increase SS amplitude and boost SS-SO coupling, increasing the odds of an SS locked to the slow wave which follows the first click. To evaluate the impact of stimulation phase on the subsequent SS likelihood, we set out to determine the effect of stimulation during different phases on the induced spindles. In young adults, SS likelihood increased after the click in STIM compared to SHAM, and this was particularly apparent during the rising slope of the SO corresponding to the phase interval between −54° and 133.2° (*p* < .05 after FDR correction) as shown in Figure 4a. For this phase interval, we found a mean SS likelihood of 20.8% (95% CI = 15.9% to 25.6%) in STIM compared to 8.2% (95% CI = 6.7% to 9.8%) for the same phase interval in SHAM (two-tailed Welch *t*-test, *t*(23.9) = 4.8, *p* < .001). Pairwise comparison of within stimulation condition across all evaluated click phase bins indicates that SS quantity is significantly increased when clicks were applied during the positive rising slope (−25.2° and 111.6°) compared to clicks in the falling slope (169.2° and 270.0°) (*p* < .05 after FDR correction) Figure 4b. Interestingly, we did not find defined cluster differences for the pairwise comparison for click delay bins in terms of SS likelihood in the time-locked analysis.

For young subjects, we found differences in how the phase of stimulation affects the SS measures as seen in Figure 4c. Thus, SS latency and duration were not modulated by the click phase. Nevertheless, SS RMS and amplitude showed a significant increase when the clicks occurred close to the phase of maximal SO amplitude corresponding to the interval between −18° and −3.6° and between 75.6° and 133.2° for amplitude, and to the intervals between −18° and 10.8°, and 68.4° and 133.2° for SS RMS (*p* < .05 after FDR correction).

Remarkably, it is more difficult to induce a SO-coupled spindle in the older populations. The likelihood of spindles only in the STIM condition was 4.4% (95% CI = 2.0% to 6.8%), whereas for SHAM, the average likelihood was 1.2% (95% CI = 0.3% to 2.2%). Though small, this is a significant increment (two-tailed Welch *t*-test, *t*(20.9) = 2.4; *p* = 0.027), but this was not associated with a specific SO phase as seen in Figure 4e, where the odds of finding a spindle after the stimulus click are low and not phase-dependent. Furthermore, SS latency, duration, amplitude and RMS demonstrated no phase-dependent increases (Figure 4f), and within condition analysis of inter-trial differences in this older group showed few significant changes for stimuli applied at different phases.

### Spindle incidence after stimulus is determined by click phase for young, but not older subjects

Recent studies [14, 31, 36] have suggested that SS likelihood is strongly dependent on the refractoriness of spindle-generating networks, i.e. the lag to the previous SS occurrence. However, these studies have not examined the degree to which phase of stimulation affects SS odds in the presence of SO. To evaluate these factors, we constructed a binomial logistic regression model to predict the occurrence of spindles, using the following explanatory variables: SS lag (*Lag*) and the cosine (*Cos*) and sine (*Sin*) of the click phase when the auditory stimulus was applied, whether the stimulus was applied during an ongoing SS (onSS) and the SS amplitude (SS_amp_) (see Statistic section in methods).

Table 2 presents the parameter estimates of the regression for all the events where the lag between stimulation and a subsequent spindle is <10 seconds. For young adults, a test of the full model versus a model with intercept only was statistically significant χ ^2^(3, *N* = 3293) = 12.49, *p* = .005. The outcome is primarily determined by both trigonometric components of the phase of stimulation (*Sin, Wald* χ2 (1) = 9.36, *p* = .004; *Cos, Wald χ*^*2*^ (1) = 3.91, *p* = .048), whereas the lag since prior spindle was non-significant (*Lag, Wald* χ ^2^ (1), *p* = .633). The increased power of the phase covariate is also distinguishable in Figure 4d, which presents the significant *t*-values for SS likelihood STIM vs. SHAM after FDR correction. From the estimated coefficients of this regression, the acrophase angle where the logit function reaches its maximum is at 56.8°. Interestingly, this angle of maximum odds ratio for SS occurrence is within the rising slope (−25.2° and 111.6°), which is the region of maximal response in the within stimulation condition as shown in Figure 4b.

**Table 2.** Binomial regression model for spindle likelihood in young subjects

	Estimate (β)	SE	Wald χ ^2^	df	*p*	Odds ratio	95% CI for odds ratio
*(Intercept)*	−0.80	0.05	238.78	1.00	**<.001**	0.45	0.41 to 0.50
*Lag*	0.03	0.07	0.23	1.00	.633	1.03	0.91 to 1.17
*Cos*	0.12	0.06	3.91	1.00	**.048**	1.12	1.00 to 1.28
*Sin*	0.18	0.06	8.36	1.00	**.004**	1.20	1.07 to 1.37

The binomial regression model for spindle likelihood in young subjects demonstrates that both trigonometric components of the phase of stimulation (*Cos* and *Sin*) are the main factors driving the logistic regression. *Lag*: sleep spindle (SS) lag; *Cos:* click phase cosine; *Sin:* click phase sine. *SE:* Standard error, *df:* degrees of freedom for the Wald test statistic to be compared with the chi-square distribution. Values in bold are statistically significance at alpha = 0.05.

For older subjects, the test of the full logistic regression model versus a model with only an intercept was also statistically significant χ ^2^(3, *N* = 940) = 8.42, *p* = .038, and the estimates of the regression are shown in Table 3. However, unlike results in younger participants neither trigonometric component was significant in this older group (*Sin, Wald* χ ^2^ (1) = 2.48, *p* = .115; *Cos, Wald* χ ^2^ (1) = 0.05, *p* = .830). The SS lag was the only significant covariate (*Lag, Wald* χ ^2^ (1) = 5.98, *p* = .014) with longer lags associated with higher probability of spindle associated, following the dynamics imposed by the spindle refractory period [31]. Likewise, no significant differences after FDR correction were noted when analyzing the SS likelihood by the size of difference STIM vs. SHAM as a joint function of the click phase and the SS lag.

**Table 3.** Binomial regression model for spindle likelihood in older subjects

	Estimate (β)	S.E.	Wald χ ^2^	df	*p*	Odds ratio	95% CI for odds ratio
*(Intercept)*	−1.35	0.097	196.398	1	**<.001**	0.26	0.21 to 0.31
*Lag*	−0.36	0.15	5.98	1.00	**.014**	0.70	0.52 to 0.93
*Cos*	0.02	0.12	0.05	1.00	.830	1.03	0.81 to 1.31
*Sin*	0.18	0.12	2.48	1.00	.115	1.20	0.98 to 1.50

The binomial regression model for spindle likelihood in older subjects shows that the temporal delay between spindles and the auditory click (*Lag*) is the main factor driving the logistic regression. *Lag*: sleep spindle (SS) lag; *Cos:* click phase cosine; *Sin:* click phase sine. *SE:* Standard error, *df:* degrees of freedom for the Wald test statistic to be compared with the chi-square distribution. Values in bold are statistically significance at alpha = 0.05.

Overall, these logistic regressions suggest that, while click phase is the most important factor determining whether a spindle will be elicited for young people, this is not the case in older adults. Instead, temporal lag since the last SS is the only important factor in older subjects.

## Discussion

### Summary of findings

We examined how SOs and spindles are modulated by CLAS in both young and older groups. This showed that, in both age groups, SO responses to the click stimulus are modulated by the phase of the SO at which the click is applied. Nevertheless, the SO phase at stimulation only impacted upon the stimulation of sleep spindles in the young group.

Auditory clicks can increase the amplitude of the following SO. However, our analyses demonstrate that the outcome of the stimulation is not only dependent of the state of cortical depolarization (down-state vs. up-state) when the click is applied [13, 20], but it is further specific to the phase of the ongoing SO. Additionally, we demonstrated that the optimal time period for stimulus delivery is age-dependent and is narrower in older adults. In both age groups, the increase in trough amplitude is led by the occurrence of a bigger quantity of SO events with amplitudes greater than 100 μV (Figure 2, a and c). For young subjects, these large SOs tend to occur after clicks applied during the rising slope and all the positive interval of the SO wave (Figure 2b). For older subjects, the window of opportunity in which clicks can elicit larger SOs (>100 μV) is constrained to the positive interval of the SO (Figure 2d). Importantly, in both young and older populations maximal SO responses are triggered by clicks applied at the peak SO amplitude. Therefore, the larger increment of post-stimulus trough amplitudes which occurs during critical windows suggests that SO phase modulates cortical reactivity by auditory input.

### Relationship between CLAS and auditory evoked potentials during SWS

It is well known that auditory stimuli elicit large-amplitude events during deep sleep, although the interplay between these and spontaneous sleep activity is not clear. During the N2 and N3 sleep stages, auditory inputs evoke large-amplitude late negative deflections with maximal values between 500 and 650 ms, which appear as a K-complex waveform [37]. The amplitude of these evoked events is attributed to the intensity of the acoustic stimulus, and peaks in fronto-central electrodes [37, 38]. This increasing of EEG amplitude is thought to be due to the presence of acoustic disturbance, regardless of the time when it is presented. However, our analysis suggests that the time of stimulus presentation is actually very important. Previous studies have also demonstrated that the early components of auditory evoked potentials change when stimuli are presented at different points along with the negative and positive phase of the SO in humans [39, 40]. In rats, the responses to somatosensory stimuli are modulated by SWA under anesthesia [41]. These studies suggest that wake-like periods of high neural discharge during sleep may facilitate the processing of sensory information by synchronizing larger populations of neurons with the ongoing rhythmicity of spontaneous cortical activity. Such stimuli may target the thalamus, probably via secondary ascending acoustic pathways [42]. Our findings are also congruent with previous studies of neural response to somatosensory stimulation in anesthetized cats. These studies indicate that the cortical ability to respond to peripheral stimuli during all phases of the SO is based on the intrinsic properties of thalamocortical cells and the functional properties of the medial lemniscus. This firing ability of thalamocortical cells is reduced when the stimulation is applied during the cortical down-states [43]. Thus, the potential of the stimuli to increase synchronous neural firing is maximal during wake-like intervals occurring at the peak phase of the scalp level SO [44].

### Effect of age on optimal CLAS timing

We found a marked age-related reduction in response to the incoming stimulus. Normal aging processes involve a continuous deterioration of macro-level structure and micro-level architecture of sleep, including the reduction of SO density, amplitude, and coupling with spindles [6, 45]. This decrease of the cortical capability to group larger neural populations in synchronous activity, a fact that is evident from the lower amplitude and longer period of SO in older participants, may be due to the decrease of gray matter volume presented in this population [9, 46]. As a result, we speculate that the lower response to stimulation in older participants could be caused by the diminished capacity of the cortex to react to peripheral stimuli by synchronizing the neural firing of larger populations. Our analyses localize the period during which stimulation is effective in this older population to the rising part of the positive SO deflection, a period suggested as optimal by previous studies [13, 15]. Nevertheless, for both the younger and older populations, the maximal responses in post-stimulus SO trough amplitudes occur when stimulation falls at the phase of maximal amplitude, reinforcing the idea that maximal effects of sensory inputs are obtained during times with the smallest quantity of neural silent states.

Our work also shows that the SS likelihood after stimulation is modulated by the phase of the SO in the young but not the older population. Thus, in young participants, SS likelihood can be increased by an auditory stimulus applied at most times within the rising slow wave. Nevertheless, the effect is highest when the auditory click is applied just before the maximal phase of the SO as shown in Figure 4b. For this young population, the window of opportunity to increase SS likelihood overlaps with the optimal interval for boosting of SS power and amplitude in post-stimulus events, which also occurs when stimuli are applied close to the peak phase of the SO. This finding suggests a close relationship between the increment of SS event likelihood and the induced increment of spindle band amplitude. This is also supported by our logistic regression model, which suggests that spindle generation has a stronger dependency on SO phase than on SS lag in this young cohort. By comparison, older subjects generally have a reduced SS likelihood, and although SS likelihood has been shown to be increased by CLAS in this group [15], this increase does not appear to be due to stimulation at specific SO phases. Furthermore, no changes in SS power or amplitude were related to stimulation phase in older subjects. This might be explained by the low number of SS events in each phase/delay bin in the older group, reducing the power of statistical analysis, or alternately with a genuine decrease in spindles which is characteristic in older subjects [25, 47], possibly due to reduction of gray matter [45, 48]. However, our logistic regression model suggests that SS lag may more strongly influence SS likelihood than SO phase of click stimuli in older subjects.

Our results also suggest that the phase of the ongoing SO establishes the window of opportunity where CLAS can generate subsequent SS events. Essentially, spindles are of thalamocortical origin and frequently occur phase-locked to SOs [49]. Human intra-cranial recordings suggest that convergent cortical down-states induce thalamic spindles which are then transferred back to the cortex [50]. There is also evidence that this process is regulated by periods of spindle refractoriness [31, 36]. Hence, the combined action of cortico-thalamic interplay and refractory periods could facilitate global coordination in thalamic spindle-generator networks. Thus, when an auditory stimulus arrives, this triggers even greater synchronous hyperpolarization of cortical neurons. If this occurs at the optimal SO phase, the auditory stimulus may become an indirect driver of this spindle generation process. Following this line of reasoning, the reduced extent to which external stimuli triggered spindles in our older cohort could potentially be explained by decreased capacity for neural synchronization due to lower gray matter volume [45]. If this is the case, the likelihood of auditory stimuli eliciting spindles in this age group might, therefore, be determined solely by the refractory period. This interpretation could also explain why it is difficult to entrain spindles by applying stimulation in spindle-like frequencies [26, 31] as consecutive stimuli may not be able to directly drive the thalamic pathways that promote spindle activity, whereas spindles can be entrained by using direct thalamic stimulation [51]. Nevertheless, further research is required to understand the possible thalamic drive that is indirectly caused by sensory stimulation able to increase SS likelihood [14, 52].

### Methodological limitations

We would like to acknowledge some methodological limitations of the current study that must be taken into consideration. First, as CLAS is based on the stimulation of a targeted phase, the number of events analyzed is not uniform across all phase bins. This is not problematic in bins close to the target phase, but it may affect the results of bins in the opposite phase to the target, which necessarily include a lower number of stimulation trials. Hence, one concern is that results could be driven by the unbalanced quantity of events in each bin. However, we recognized this limitation and applied a statistical methodology that restrains the effect of unbalanced samples across different bins. To this end, we generated representative distributions for each bin that fit the original data while increasing the statistical power and balancing the effect size across bins using the applied Monte Carlo method. Nevertheless, we are still cautious in interpreting null-effects of the stimulus where the number of events is small, e.g. in the SO trough.

A second limitation relates to how the second click could affect the characteristics of post-stimulus spindles. Because it often fell within the window during which spindles were examined, the second click could theoretically have impacted upon the characteristics of the upcoming post-stimulus spindles. However, several characteristics of CLAS lead us to believe that the second click had a negligible impact on the spindle effect. First, the CLAS-related spindle increment is restricted to the first click, independent of the number of consecutive stimuli [14]. Second, this spindle increment occurs reliably on the SO occurring immediately after the first click [13, 14, 16]. Therefore, the selected area for spindle detection is appropriate to evaluate the effect of the click on spindle activity.

A third limitation relates to phase analysis, which may suggest a theoretical sinusoidal shape of the SO wave. We would like to clarify that we do not consider the SO as a perfectly defined sine wave; however, we take advantage of the mathematical properties of the sine wave in considering the analytical signal of the SWA and, therefore, its phase description. Here, it is important to emphasize that the phase description is only meaningful for mono-component time series [27, 28], and the interpretation of the phase of highest amplitude should be taken with care in trials containing multiple peaks in the SWA range.

## Conclusions

In sum, we investigated the phase-dependent outcomes of CLAS in small temporal windows and identified the optimal ranges for acoustic stimulation to evoke the highest effect on SOs and SS in both young and middle-aged populations. We found that optimal timing is at the peak of the SO, and the window of opportunity to optimally stimulate older subjects is narrower than for the young population. Consequently, we suggest implementing adaptative CLAS algorithms that can better determine the timing for stimulation of SO in older populations. We speculate that the windows of opportunity to generate optimal responses could be defined by the wake-like intervals in which sensory perturbation can recruit large thalamocortical populations in synchronous hyperpolarization.

## References

[1] Iber C, et al. The AASM Manual for the Scoring of Sleep and Associated Events: Rules Terminology and Technical Specifications, 1st ed. Westchester, IL: American Academy of Sleep Medicine; 2007:59.

[2] Rasch B, et al About sleep’s role in memory. Physiol Rev. 2013;93(2):681–766. 23589831 10.1152/physrev.00032.2012 PMC3768102

[3] Besedovsky L, et al Auditory closed-loop stimulation of EEG slow oscillations strengthens sleep and signs of its immune-supportive function. Nat Commun. 2017;8(1):1984. 29215045 10.1038/s41467-017-02170-3 PMC5719447

[4] Xie L, et al. Sleep drives metabolite clearance from the adult brain. Science. 2013;342(6156):373–377. 24136970 10.1126/science.1241224 PMC3880190

[5] Ohayon MM, et al Meta-Analysis of quantitative sleep parameters from childhood to old age in healthy individuals: developing normative sleep values across the human lifespan. Sleep. 2004;27(7):1255–1273. 15586779 10.1093/sleep/27.7.1255

[6] Mander BA, et al Sleep and human aging. Neuron. 2017;94(1):19–36. 28384471 10.1016/j.neuron.2017.02.004 PMC5810920

[7] Scullin MK, et al Sleep, cognition, and normal aging: integrating a half century of multidisciplinary research. Perspect Psychol Sci. 2015;10(1):97–137. 25620997 10.1177/1745691614556680 PMC4302758

[8] Scullin MK Sleep, memory, and aging: the link between slow-wave sleep and episodic memory changes from younger to older adults. Psychol Aging. 2013;28(1):105–114. 22708533 10.1037/a0028830 PMC3532961

[9] Mander BA, et al White matter structure in older adults moderates the benefit of sleep spindles on motor memory consolidation. J Neurosci. 2017;37(48):11675–11687. 29084867 10.1523/JNEUROSCI.3033-16.2017 PMC5707766

[10] Massimini M, et al Triggering sleep slow waves by transcranial magnetic stimulation. Proc Natl Acad Sci U S A. 2007;104(20):8496–8501. 17483481 10.1073/pnas.0702495104 PMC1895978

[11] Marshall L, et al Transcranial direct current stimulation during sleep improves declarative memory. J Neurosci. 2004;24(44):9985–9992. 15525784 10.1523/JNEUROSCI.2725-04.2004 PMC6730231

[12] Ketz N, et al Closed-Loop slow-wave tacs improves sleep-dependent long-term memory generalization by modulating endogenous oscillations. J Neurosci. 2018;38(33):7314–7326. 30037830 10.1523/JNEUROSCI.0273-18.2018 PMC6596034

[13] Ngo HV, et al Auditory closed-loop stimulation of the sleep slow oscillation enhances memory. Neuron. 2013;78(3):545–553. 23583623 10.1016/j.neuron.2013.03.006

[14] Ngo HV, et al Driving sleep slow oscillations by auditory closed-loop stimulation-a self-limiting process. J Neurosci. 2015;35(17):6630–6638. 25926443 10.1523/JNEUROSCI.3133-14.2015 PMC4412888

[15] Papalambros NA, et al Acoustic enhancement of sleep slow oscillations and concomitant memory improvement in older adults. Front Hum Neurosci. 2017;11:109. 28337134 10.3389/fnhum.2017.00109 PMC5340797

[16] Ong JL, et al Auditory stimulation of sleep slow oscillations modulates subsequent memory encoding through altered hippocampal function. Sleep. 2019;42(2). doi:10.1093/sleep/zsy240. 10.1093/sleep/zsy240 PMC6369722 30535413

[17] Grimaldi D, et al Strengthening sleep–autonomic interaction via acoustic enhancement of slow oscillations. Sleep. 2019;42(5). doi:10.1093/sleep/zsz036 10.1093/sleep/zsz036 PMC7729207 30753650

[18] Leminen M, et al Enhanced memory consolidation via automatic sound stimulation during non-rem sleep. Sleep. 2017;40(3). doi:10.1093/sleep/zsx003 10.1093/sleep/zsx003 PMC5806588 28364428

[19] Santostasi G, et al Phase-locked loop for precisely timed acoustic stimulation during sleep. J Neurosci Methods. 2016;259:101–114. 26617321 10.1016/j.jneumeth.2015.11.007 PMC5169172

[20] Schabus M, et al The fate of incoming stimuli during nrem sleep is determined by spindles and the phase of the slow oscillation. Front Neurol. 2012;3:40. 22493589 10.3389/fneur.2012.00040 PMC3319907

[21] Batterink LJ, et al Phase of spontaneous slow oscillations during sleep influences memory-related processing of auditory cues. J Neurosci. 2016;36(4):1401–1409. 26818525 10.1523/JNEUROSCI.3175-15.2016 PMC4728733

[22] Riedner BA, et al Sleep homeostasis and cortical synchronization: III. A high-density EEG study of sleep slow waves in humans. Sleep. 2007;30(12):1643–1657. 18246974 10.1093/sleep/30.12.1643 PMC2276133

[23] Clemens Z, et al Temporal coupling of parahippocampal ripples, sleep spindles and slow oscillations in humans. Brain. 2007;130(Pt 11):2868–2878. 17615093 10.1093/brain/awm146

[24] Warby SC, et al Sleep-spindle detection: crowdsourcing and evaluating performance of experts, non-experts and automated methods. Nat Methods. 2014;11(4):385–392. 24562424 10.1038/nmeth.2855 PMC3972193

[25] Purcell SM, et al Characterizing sleep spindles in 11,630 individuals from the National Sleep Research Resource. Nat Commun. 2017;8(May):15930. 28649997 10.1038/ncomms15930 PMC5490197

[26] Ngo HV, et al Insights on auditory closed-loop stimulation targeting sleep spindles in slow oscillation up-states. J Neurosci Methods. 2019;316:117–124. 30194953 10.1016/j.jneumeth.2018.09.006

[27] Boashash B Estimating and interpreting the instantaneous frequency of a signal. I. Fundamentals. Proc IEEE. 1992;80(4):540–568.

[28] Chavez M, et al Towards a proper estimation of phase synchronization from time series. J Neurosci Methods. 2006;154(1-2):149–160. 16445988 10.1016/j.jneumeth.2005.12.009

[29] Berens P CircStat: a MATLAB toolbox for circular statistics. J Stat Softw. 2009;31(10):1–21.

[30] Benjamini Y, et al The control of the false discovery rate in multiple testing under dependency. Ann Stat. 2001;29(4):1165–1188.

[31] Antony JW, et al Sleep spindle refractoriness segregates periods of memory reactivation. Curr Biol. 2018;28(11):1736–1743.e4. 29804809 10.1016/j.cub.2018.04.020 PMC5992601

[32] Mardia KV Linear-Circular correlation coefficients and rhythmometry. Biometrika. 1976;63(2):403.

[33] Mardia KV, et al A model for cylindrical variables with applications. J R Stat Soc Ser B. 1978;40(2):229–233.

[34] Jammalamadaka SR, Sengupta A. Series on Multivariate Analysis: Volume 5 Topics in Circular Statistics, vol 5 Singapore. World Scientific Publishing Co Pte Ltd; 2001.

[35] Al-Daffaie K, et al Logistic regression for circular data. In: AIP Conference Proceedings: The 3rd ISM International Statistical Conference 2016 (ISM III). Vol 1842 2017:030022.

[36] Lecci S, et al Coordinated infraslow neural and cardiac oscillations mark fragility and offline periods in mammalian sleep. Sci Adv. 2017;3(2):e1602026. 28246641 10.1126/sciadv.1602026 PMC5298853

[37] Colrain IM, et al The N550 component of the evoked K-complex: a modality non-specific response? J Sleep Res. 1999;8(4):273–280. 10646167 10.1046/j.1365-2869.1999.00163.x

[38] Riedner BA, et al Temporal dynamics of cortical sources underlying spontaneous and peripherally evoked slow waves. Prog Brain Res. 2011;193:201–218. 21854964 10.1016/B978-0-444-53839-0.00013-2 PMC3160723

[39] Massimini M EEG slow (~1 hz) waves are associated with nonstationarity of thalamo-cortical sensory processing in the sleeping human. J Neurophysiol. 2002;89(3):1205–1213. 10.1152/jn.00373.2002 12626608

[40] Rector DM, et al Mechanisms underlying state dependent surface-evoked response patterns. Neuroscience. 2009;159(1):115–126. 19154778 10.1016/j.neuroscience.2008.11.031 PMC2706571

[41] Haslinger R, et al Analysis of LFP phase predicts sensory response of barrel cortex. J Neurophysiol. 2006;96(3):1658–1663. 16775200 10.1152/jn.01288.2005

[42] Bellesi M, et al Enhancement of sleep slow waves: underlying mechanisms and practical consequences. Front Syst Neurosci. 2014;8:208. 25389394 10.3389/fnsys.2014.00208 PMC4211398

[43] Rosanova M, et al Neuronal mechanisms mediating the variability of somatosensory evoked potentials during sleep oscillations in cats. J Physiol. 2005;562(Pt 2):569–582. 15528249 10.1113/jphysiol.2004.071381 PMC1665518

[44] Nir Y, et al Regional slow waves and spindles in human sleep. Neuron. 2011;70(1):153–169. 21482364 10.1016/j.neuron.2011.02.043 PMC3108825

[45] Helfrich RF, et al Old brains come uncoupled in sleep: slow wave-spindle synchrony, brain atrophy, and forgetting. Neuron. 2018;97(1):221–230.e4. 29249289 10.1016/j.neuron.2017.11.020 PMC5754239

[46] Saletin JM, et al Structural brain correlates of human sleep oscillations. Neuroimage. 2013;83:658–668. 23770411 10.1016/j.neuroimage.2013.06.021 PMC4263481

[47] Clawson BC, et al Form and function of sleep spindles across the lifespan. Neural Plast. 2016;2016:6936381. 27190654 10.1155/2016/6936381 PMC4848449

[48] Landolt HP, et al Effect of age on the sleep EEG: slow-wave activity and spindle frequency activity in young and middle-aged men. Brain Res. 1996;738(2):205–212. 8955514 10.1016/s0006-8993(96)00770-6

[49] Mölle M, et al Fast and slow spindles during the sleep slow oscillation: disparate coalescence and engagement in memory processing. Sleep. 2011;34(10):1411–1421. 21966073 10.5665/SLEEP.1290 PMC3174843

[50] Mak-McCully RA, et al Coordination of cortical and thalamic activity during non-REM sleep in humans. Nat Commun. 2017;8:15499. 28541306 10.1038/ncomms15499 PMC5458505

[51] Latchoumane CFV, et al Thalamic spindles promote memory formation during sleep through triple phase-locking of cortical, thalamic, and hippocampal rhythms. Neuron. 2017;95(2):424–435.e6. 28689981 10.1016/j.neuron.2017.06.025

[52] Lustenberger C, et al High-density EEG characterization of brain responses to auditory rhythmic stimuli during wakefulness and NREM sleep. Neuroimage. 2018;169:57–68. 29217404 10.1016/j.neuroimage.2017.12.007 PMC5856588

